# Venetoclax and Azacitidine in the Treatment of Blastic Plasmacytoid Dendritic Cell Neoplasm Refractory to Conventional Therapy

**DOI:** 10.7759/cureus.33109

**Published:** 2022-12-29

**Authors:** Farhan Azad, Jiahua Zhang, Clive J Miranda, Matthew Gravina

**Affiliations:** 1 Internal Medicine, University at Buffalo, Buffalo, USA; 2 Internal Medicine, Mercy Hospital St. Louis, St. Louis, USA; 3 Hematology and Medical Oncology, University at Buffalo, Buffalo, USA

**Keywords:** cd123, pivekimab, azacitidine, venetoclax, tagraxofusp, bpdcn

## Abstract

Blastic plasmacytoid dendritic cell neoplasm is a rare and aggressive hematological malignancy associated with poor prognosis and limited treatment options. No guideline-directed therapy existed until the approval of tagraxofusp in 2018 by the Food and Drug Administration. Multiple clinical trials are undergoing as treatment options continue to evolve. We report a case refractory to tagraxofusp and pivekimab sunirine with subsequent remission achieved on venetoclax and azacitidine therapy.

## Introduction

Blastic plasmacytoid dendritic cell neoplasm (BPDCN) is an aggressive condition involving the clonal proliferation of immature precursor plasmacytoid dendritic cells (pDCs). It is a rare disease that occurs in elderly known to disseminate rapidly with high rates of relapse and poor prognosis [1]. It accounts for 0.44% of all hematological malignancies [2]. Patients present with skin lesions, although extracutaneous involvement including lymphadenopathy and hepatosplenomegaly is common. Central nervous system (CNS) involvement is frequent at both diagnosis and relapse, with incidence ranging from 9% to 26% [3]. Sixty to ninety percent of patients have peripheral blood and bone marrow involvement [4]. Previously classified under acute myeloid leukemia and related precursor neoplasms by the World Health Organization in 2008, BPDCN was reclassified as a separate myeloid malignancy in 2016 as we continue to learn about this disease [5]. We present a case of BPDCN refractory to typical chemotherapy, treated well with venetoclax and azacitidine, showing excellent response two months after therapy.

## Case presentation

A 70-year-old Caucasian male with no significant past medical history presented with multiple persistent painless new skin lesions on his back, increasing in number and size over a month. The patient denied any history of smoking, drinking alcohol, or using any recreational substances. He was not taking any home medications. On exam, a 4cm x 5cm atrophic patch was noted on the right back. The left frontal scalp had a 4 cm x 5cm translucent tumor which was 0.5 cm in height with central necrosis. The right mid crown was keratotic with a thin red plaque of about 1.5 cm, with faded lesions to the chest, anterior torso, and back. No palpable hepatosplenomegaly or lymphadenopathy was noted. Initial laboratory investigations before venetoclax and azacitidine therapy are shown in Table 1.

**Table 1:** Initial laboratory investigations MCV: Mean corpuscular volume; GFR: glomerular filtration rate; BUN: blood urea nitrogen

Laboratory investigations	Results	Reference Range
Complete blood count		
White blood cells	5.2x10^9^/L	4.0-10.5x10^9^/L
Hemoglobin	10.5 g/dL	12.0-16.0 g/dL
Hematocrit	31.8%	37.0-47.0%
MCV	84.7 fL	80.0-100.0 fL
Platelet count	240x10^9^/L	140x10^9^-400x10^9^/L
Basic metabolic panel		
Sodium level	139 mmol/L	133-147 mmol/L
Potassium level	4.0 mmol/L	3.5-5.6 mmol/L
Chloride	109 mmol/L	96-110 mmol/L
Carbon dioxide	25 mmol/L	20-32 mmol/L
Calcium level	9.1 mg/dL	6.3-11.9 mg/dL
GFR	>60 mL/min/1.73 m2	>=60 mL/min/1.73 m2
BUN	13 mg/dL	5-27 mg/dL
Creatinine	0.91 mg/dL	0.40-1.40 mg/dL
Glucose level	95 mg/dL	60-100 mg/dL

**Figure 1:**
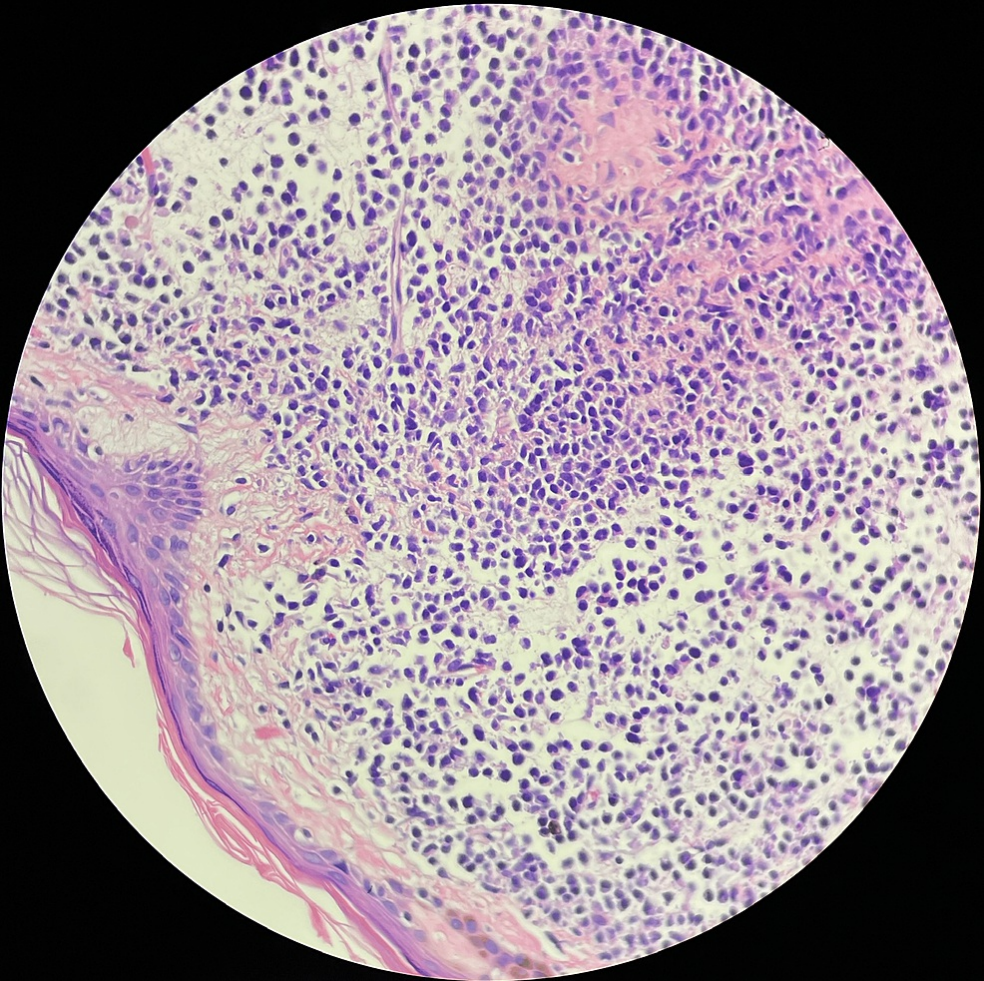
Skin biopsy showing dense dermal and subcutaneous malignant hematologic infiltrate composed of monotonous medium/large cells with variable nuclear irregularities, blastoid chromatin, and granular cytoplasm

Skin biopsy revealed dense dermal and subcutaneous malignant hematologic infiltrate composed of monotonous medium/large cells with variable nuclear irregularities, blastoid chromatin, and granular cytoplasm (Figure 1).

**Figure 2:**
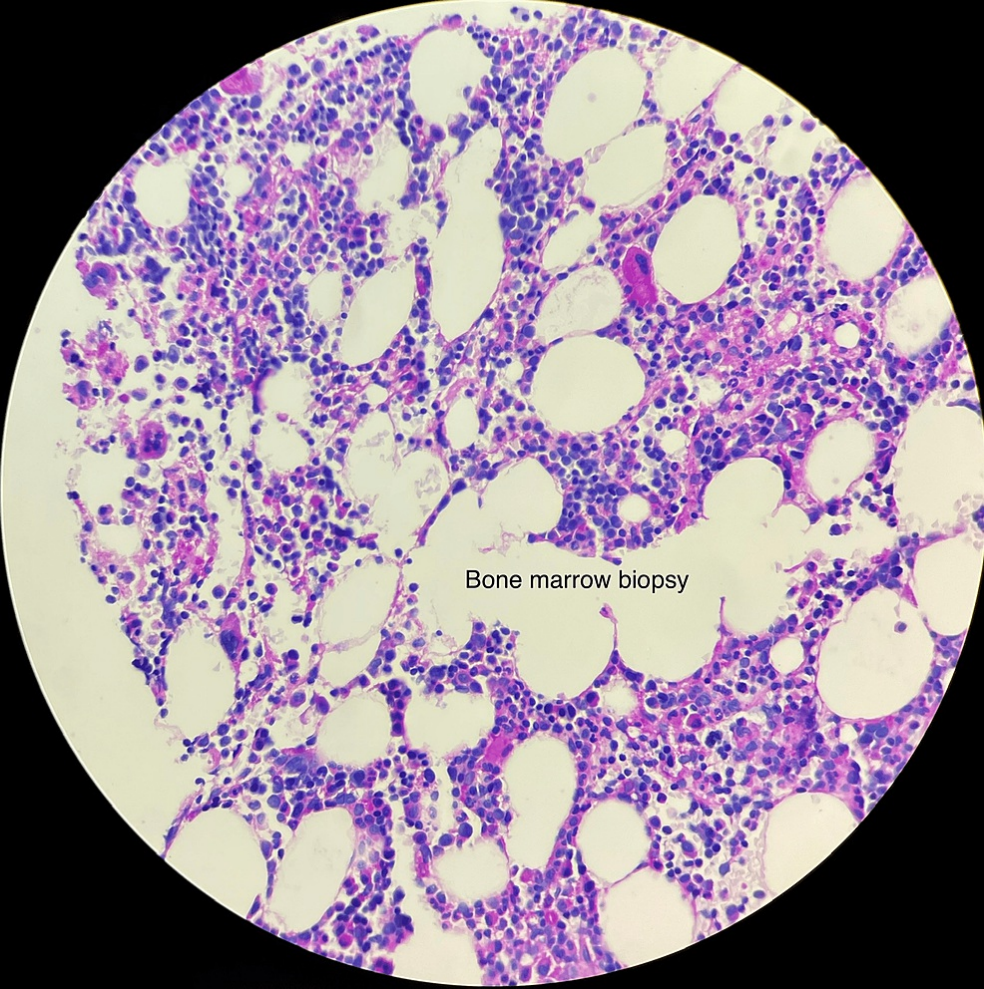
Bone marrow biopsy showing hypercellular marrow (around 70%) with trilineage hematopoiesis and malignant infiltrate consistent with BPDCN involving 5-10% of cellularity BPDCN: Blastic plasmacytoid dendritic cell neoplasm

**Figure 3:**
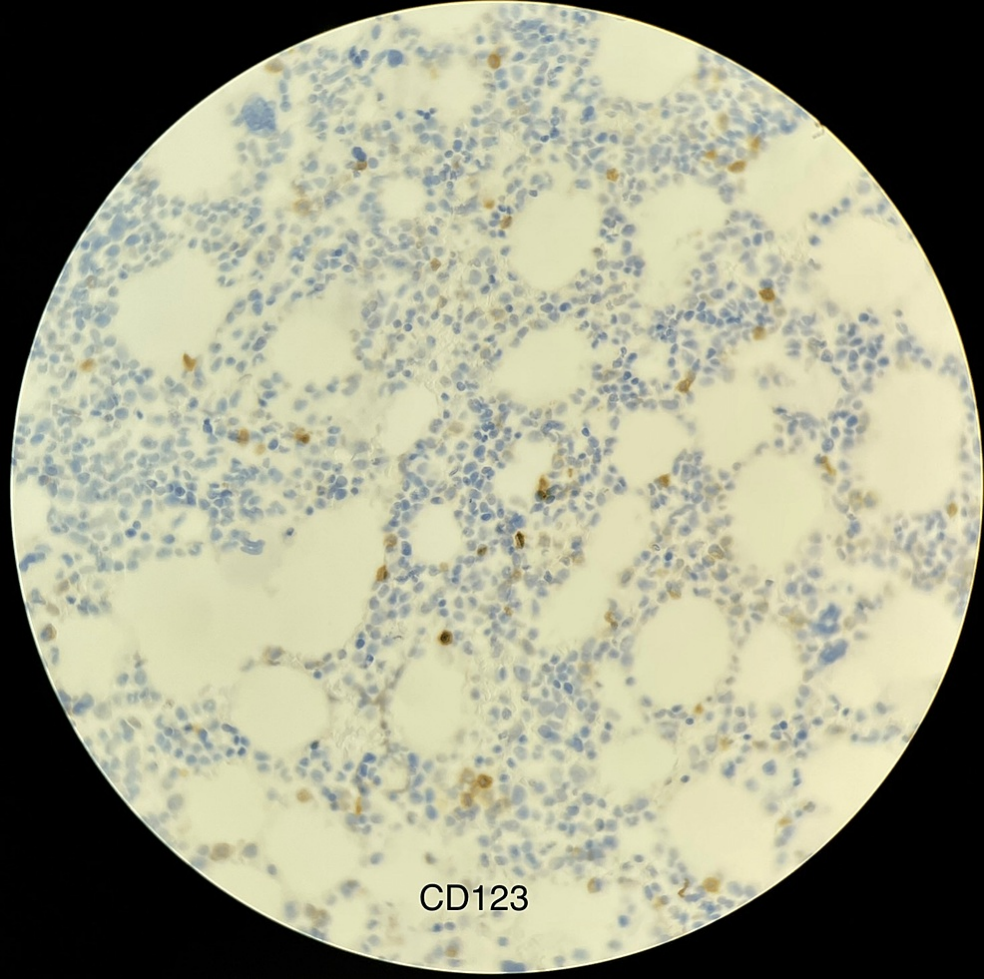
Clusters of plasmacytoid dendritic cells positive for CD123 immunostaining

The cells were positive for CD2, CD4, CD56, CD45, CD123, and BCL2 and negative for BCL6, Epstein-Barr-encoding region (EBER), myeloperoxidase (MPO), and lysozyme. Molecular studies for t-cell receptor gamma, immunoglobulin heavy chain, and immunoglobulin kappa light chain were negative. Bone marrow biopsy (BMB) revealed hypercellular marrow (around 70%) with trilineage hematopoiesis and malignant infiltrate consistent with BPDCN involving 5-10% of cellularity (Figure 2). Clusters of pDCs were seen which were positive for CD123 immunostaining (Figure 3).

 

**Figure 4:**
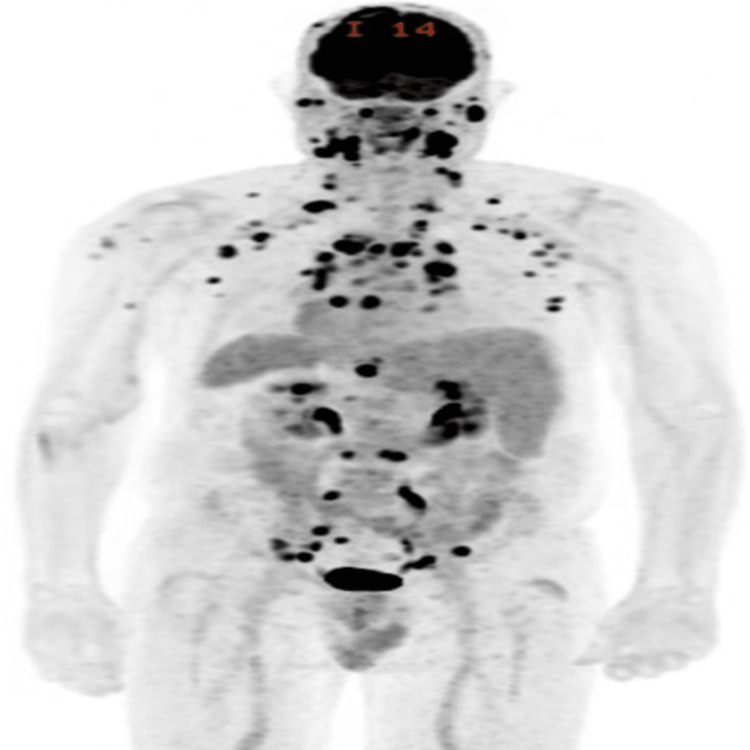
PET scan of the patient with biopsy-confirmed BPDCN after failure of two lines of therapy showing multiple FDG-avid sites of disease PET: Positron emission tomography; BPDCN: blastic plasmacytoid dendritic cell neoplasm; FDG: F-fluorodeoxyglucose

Peripheral blood smear showed normocytic normochromic anemia. Initial positron emission tomography (PET) scan revealed metabolically active malignancy involving lymph node chains throughout the neck/chest/abdomen/pelvis, subcutaneous soft tissue and skin lesions, spleen, and left humerus (Figure 4).

**Figure 5:**
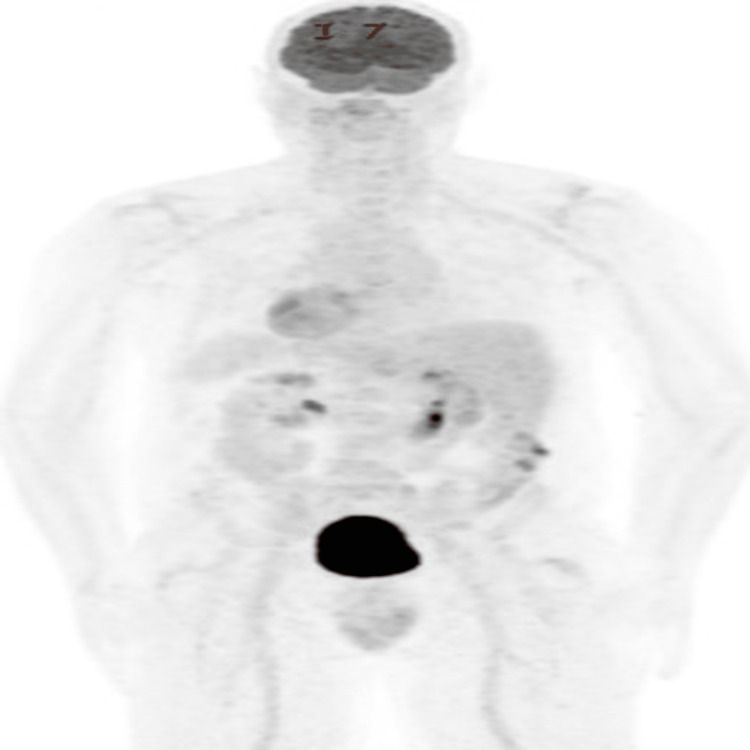
PET scan of the patient with refractory BPDCN after two cycles of venetoclax and azacitidine showing a complete metabolic response PET: Positron emission tomography; BPDCN: blastic plasmacytoid dendritic cell neoplasm

Lesions enlarged despite three cycles of tagraxofusp. He was started on pivekimab sunirine as per clinical trial for CD123-positive malignancies. Neither tagraxofusp nor pivekimab sunirine elicited any side effects in our patient, although they proved ineffective for his skin lesions. Lumbar puncture (LP) showed rare, atypical mononuclear cells suspicious for 0.5% involvement by BPDCN by flow cytometry. He was started on weekly intrathecal chemotherapy (IT) with cytarabine/hydrocortisone. Repeat LPs after four weeks of IT were negative. After a cycle of pivekimab sunirine, skin lesions continued to worsen, and then they stopped. He was given two cycles of venetoclax with azacitidine (Ven/Aza) at 100 mg and 50 mg/m^2^ respectively with resolution of his skin lesions. Ven/Aza was initiated given BCL-2 positivity seen in the patient’s laboratory values, and no side effects were observed. Restaging BMB two months later revealed normocellular marrow (40% cellularity) with trilineage maturing hematopoiesis and no residual BPDCN. A repeat PET scan two months later showed a great metabolic response to therapy (Figure 5).

Subsequent laboratory investigations after two cycles of venetoclax and azacitidine therapy are shown in Table 2.

**Table 2:** Subsequent laboratory investigations MCV: Mean corpuscular volume; GFR: glomerular filtration rate; BUN: blood urea nitrogen

Laboratory investigations	Results	Reference Range
Complete blood count		
White blood cells	3.0x10^9^/L	4.0-10.5x10^9^/L
Hemoglobin	9.1 g/dL	12.0-16.0 g/dL
Hematocrit	28.0%	37.0-47.0%
MCV	77.8 fL	80.0-100.0 fL
Platelet count	325x10^9^/L	140x10^9^-400x10^9^/L
Basic metabolic panel		
Sodium level	139 mmol/L	133-147 mmol/L
Potassium level	4.3 mmol/L	3.5-5.6 mmol/L
Chloride	107 mmol/L	96-110 mmol/L
Carbon dioxide	28 mmol/L	20-32 mmol/L
Calcium level	9.3 mg/dL	6.3-11.9 mg/dL
GFR	>60 mL/min/1.73 m2	>=60 mL/min/1.73 m2
BUN	13 mg/dL	5-27 mg/dL
Creatinine	1.10 mg/dL	0.40-1.40 mg/dL
Glucose level	81 mg/dL	60-100 mg/dL

Currently, he was reluctant to pursue stem cell transplantation (SCT).

## Discussion

The diagnosis was made based on biopsy and immunohistochemical findings. BPDCN characteristically expresses CD4, CD56, and CD123 in the absence of other myeloid leukemia markers including MPO and lysozyme, as seen in our case [6]. Most cases of BPDCN are diagnosed with a skin biopsy [7]. For our patient, both skin and bone marrow biopsies corroborated with the diagnosis of BPDCN. Distinguishing other common malignancies with cutaneous involvement is also important. Cutaneous t-cell lymphoma usually lacks CD56 expression, while extra-nodal natural killer cell malignancies are typically EBER-positive [8]. With positive CD56 and negative EBER expression, other malignancies with cutaneous manifestations were ruled out in our case.

Until 2018, there was no consensus on approved therapies for BPDCN. It was treated with induction chemotherapy used in other acute leukemias followed by SCT. Unfortunately, frequent relapse rates were seen [9]. In 30% of cases, relapse happens in the CNS. Prophylactic intrathecal therapy and SCT have been shown to have better outcomes [10]. While our patient did not have any evidence of CNS disease, he continued to decline SCT, further necessitating the importance of determining the optimal medication regimen. In 2018, the Food and Drug Administration approved tagraxofusp, a CD123-directed cytotoxin as the first-line treatment based on trial STML-401-0114. Tagraxofusp showed an overall response rate of 90% in previously untreated patients and a 72% rate of complete response with minimal residual skin abnormality (CRc). Adverse reactions included nausea, peripheral edema, and weight increase along with fatal side effects, most notably capillary leak syndrome (CLS) [11]. Despite the efficacy data in the trial, our patient’s skin lesions persisted with three cycles of tagraxofusp. Pivekimab sunirine is a CD123-targeting antibody-drug conjugate approved in 2020 for breakthrough therapy for relapsed/refractory BPDCN. Trial NCT03386513 is currently undergoing to assess its safety and efficacy. Three of three patients have so far shown a complete response of up to ten months without a transplant, and no CLS has been reported [12]. Unfortunately, the result was the same, and our patient continued to have worsening skin lesions after a cycle of pivekimab sunirine.

Studies reveal that BPDCN is dependent on the antiapoptotic protein BCL-2. Venetoclax, a BCL-2 inhibitor, is currently being explored as a treatment modality. Monotherapy with venetoclax has shown promise, with patients showing partial and complete responses in case reports [13]. A combination of venetoclax and hypomethylating agents (HMAs) is also being investigated. The primary mechanism for resistance to tagraxofusp is hypermethylation of the diphthamide biosynthesis 1 (DPH1) gene. HMAs such as azacitidine work to reverse this resistance. Case reports show that both venetoclax and azacitidine are associated with short-lived responses and extending the response might require a combination with other modalities and further clinical trials. Despite remission achieved in our patient, progressive cytopenia is a concern both with the course of BPDCN and with Ven/Aza combination therapy [14]. Given the reduction in white blood cell count and hemoglobin following Ven/Aza therapy in our patient, he will need regular monitoring with weekly cycles of chemotherapy. Trials investigating the effects of venetoclax either as a single agent (NCT03485547) or in combination with tagraxofusp (NCT03113643) are ongoing [15-16].

Allogenic hematopoietic cell transplantation (allo-HCT) is typically offered to eligible patients who achieve remission with initial therapy. While no clinical trials exist comparing outcomes in transplantation to non-transplant therapies, retrospective analyses and a meta-analysis have shown three-year overall survival rates of more than 60% for patients transplanted in first complete remission (CR1) and more than 40% for patients beyond CR1 [17]. While allo-HCT offers the possibility of prolonged remission and a possible cure, the relapse rate remains high ranging from 30% to 40% [18]. Our patient achieved a complete metabolic response with two cycles of venetoclax and azacitidine. However, given the tenuous course of BPDCN, our patient will need regular follow-up and ongoing discussion for allo-HCT.

## Conclusions

BPDCN is an uncommon condition with no consensus standard treatment regimen until recently. While therapies have shown promise in clinical trials, it is a difficult disease to treat due to its aggressive nature and considering that many of the trials are still undergoing. Our patient with BPDCN resistant to conventional therapy achieved complete remission with venetoclax and azacitidine combination regimen. Given the many unknowns about the disease and its evolving therapy options, patients require regular follow-up and discussion about SCT if eligible.
